# Combination of an Oxindole Derivative with (−)-β-Elemene Alters Cell Death Pathways in FLT3/ITD^+^ Acute Myeloid Leukemia Cells

**DOI:** 10.3390/molecules28135253

**Published:** 2023-07-06

**Authors:** Jowaher Alanazi, Onur Bender, Rumeysa Dogan, Jonaid Ahmad Malik, Arzu Atalay, Taha F. S. Ali, Eman A. M. Beshr, Ahmed M. Shawky, Omar M. Aly, Yasir Nasser H. Alqahtani, Sirajudheen Anwar

**Affiliations:** 1 Department of Pharmacology and Toxicology, College of Pharmacy, University of Hail, Hail 55476, Saudi Arabia; 2 Biotechnology Institute, Ankara University, Ankara 06135, Turkey; 3 Department of Biomedical Engineering, Indian Institute of Technology Ropar, Rupnagar 140001, India; 4 Department of Medicinal Chemistry, Faculty of Pharmacy, Minia University, Minia 61519, Egypt; 5 Science and Technology Unit (STU), Umm Al-Qura University, Makkah 21955, Saudi Arabia; 6 Department of Medicinal Chemistry, Faculty of Pharmacy, Port Said University, Port Said 42511, Egypt; 7 MOI Clinics, Security Forces Hospital, Riyadh 11481, Saudi Arabia

**Keywords:** acute myeloid leukemia, FLT3, ITD, β-elemene, oxindole, MV4-11, synergyfinder

## Abstract

Acute myeloid leukemia (AML) is one of the cancers that grow most aggressively. The challenges in AML management are huge, despite many treatment options. Mutations in FLT3 tyrosine kinase receptors make the currently available therapies less responsive. Therefore, there is a need to find new lead molecules that can specifically target mutated FLT3 to block growth factor signaling and inhibit AML cell proliferation. Our previous studies on FLT3-mutated AML cells demonstrated that β-elemene and compound **5a** showed strong inhibition of proliferation by blocking the mutated FLT3 receptor and altering the key apoptotic genes responsible for apoptosis. Furthermore, we hypothesized that both β-elemene and compound **5a** could be therapeutically effective. Therefore, combining these drugs against mutated FLT3 cells could be promising. In this context, dose–matrix combination-based cellular inhibition analyses, cell morphology studies and profiling of 43 different apoptotic protein targets via combinatorial treatment were performed. Our studies provide strong evidence for the hypothesis that β-elemene and compound **5a** combination considerably increased the therapeutic potential of both compounds by enhancing the activation of several key targets implicated in AML cell death.

## 1. Introduction

Cancer is one of the deadliest diseases worldwide and has always been challenging. It is a very heterogeneous disease, with more than 200 species, and its causes are very complex. Hallmarks such as proliferation, invasion, and metastasis abilities; angiogenesis; and resistance to drugs are the main features of cancer. In addition, by influencing biogenesis, it can use all processes to its advantage, such as epithelial mesenchymal transition or microRNAs. A wide range of therapeutics including small inhibitory molecules, smart drugs, repurposed drugs, natural products, targeted agents such as siRNA, or monoclonal antibodies address this multi-mechanistic disease. Both diagnosis and treatment processes vary depending on the type of cancer and its origin [1,2,3,4,5,6,7,8]. Acute myeloid leukemia (AML) is an aggressive cancer that usually avoids terminal differentiation [9]. The American Cancer Society (ACS) reported that around 59,610 new cases of AML and 23,710 deaths due to AML occur each year in the United States alone [10]. In the newly diagnosed ⅓ of AML cases, there are reports of interruption of differentiation-inducing transcription factors [9,11,12]. It is well known that the occurrence of multiple mutations leads to carcinogenesis. In AML, two mutations occur, one for promoting proliferation and the other for blocking differentiation through growth-factor-signaling pathways [13]. The most common type of mutation in AML is internal tandem duplication mutations of FLT3 [13,14]. Mutations in FLT3 constitutively activate the growth factor receptors, leading to cancer growth [15]. For targeting AML, one of the most important targets is the FLT3 oncoprotein. FLT3 develops various mutations during late AML pathogenesis [16]. FLT3-ITD mutations have a negative prognostic effect on patient outcome [9].

It is reported that FLT3 inhibitors have demonstrated promising results in clinical settings with minimal side effects [17,18]. The standard treatment option includes chemotherapy and a bone marrow (BM) transplant [19]. However, intensive chemotherapy followed by BM transplantation may result in various complications, particularly in older people. According to epidemiological data, AML mostly affects adults over 60 because with advancing age, frailty and comorbidities make subjects susceptible to AML [20]. There is always a need for better therapeutics for treating AML subjects. A FLT3 mutation is found in 30% of AML subjects, and can be either FLT3-ITD (internal tandem duplication mutations) or FLT3-TKD (tyrosine kinase point mutations) [21]. FLT3-ITD are considered the most important target in AML as they have been associated with poor outcomes in AML [22]. FLT3-ITD and FLT3-TKD mutations lead to the disruption of a regulated signaling cascade, causing uncontrolled signaling via the PI3K, STAT5, and ERK pathways that leads to AML cancer cell proliferation and stem cell transformation [16]. Therefore, targeting FLT3 mutations in AML can disrupt the AML transduction pathways. These FLT3 oncoproteins are very important for AML survival [16]. It is reported from various studies that blocking FLT3 in AML leads to apoptosis in vitro and in vivo [23]. Presently, several drugs are utilized against AML based on the rationale of targeting FLT3 oncoproteins [23].

In earlier studies, drugs combined against cancer have led to positive results in cancer patients [1]. Therefore, using a combination of drugs to target a particular disease is a promising step to boost therapeutic outcomes in cancer patients. The combined drugs may have enhanced efficacy compared to a single-anticancer-agent approach, as different drugs act through different signaling pathways. Using a combined drugs approach can reduce the chances of drug resistance, decrease the metastatic potential and tumor growth, reduce the number of cancer stem cells, induce apoptosis, and arrest mitotically active cells [24]. We carried out some studies on AML utilizing β-elemene against FLT3, and we observed strong inhibition of the AML cell proliferation rate [25]. β-elemene is a wide-spectrum anticancer agent, a sesquiterpene, used in traditional Chinese medicine. It is one of three isomers, with the others being δ-elemene and α-elemene [26]. β-elemene is a promising agent with potent anticancer against several different cancer types. β-elemene has been found to act through various pathways, such as blocking various cell survival pathways and promoting cell death pathways. Some studies have revealed that β-elemene induces apoptosis in cancer cells [26]. Furthermore, we designed and synthesized various synthetic oxindole-based compounds targeting FLT3 that showed positive results against AML [14]. Compound **5a**, an oxindole-based compound, was one of the most potent drugs and showed remarkable activity, specifically against FLT3 ITD-mutated AML cells. Oxindole-based compounds are classified as endogenous hetero-aromatic compounds with wide biological activities. They have been used as scaffolds for designing biological drug compounds [27].

Our previous results utilizing β-elemene and **5a** demonstrated potent effects against FLT3/ITD^+^ AML cells [14,25]. In this study, a synergistic pharmacological inhibition approach against FLT3-mutated AML cells was investigated using the combination of **5a** and β-elemene. Our results in this study show that this combination approach altered the cell death pathways in FLT3-ITD-mutated acute myeloid leukemia cells.

## 2. Results

### 2.1. 4 × 4 Dose Matrix Cellular Inhibition Profile of β-Elemene and Compound **5a**

**Figure 1:**
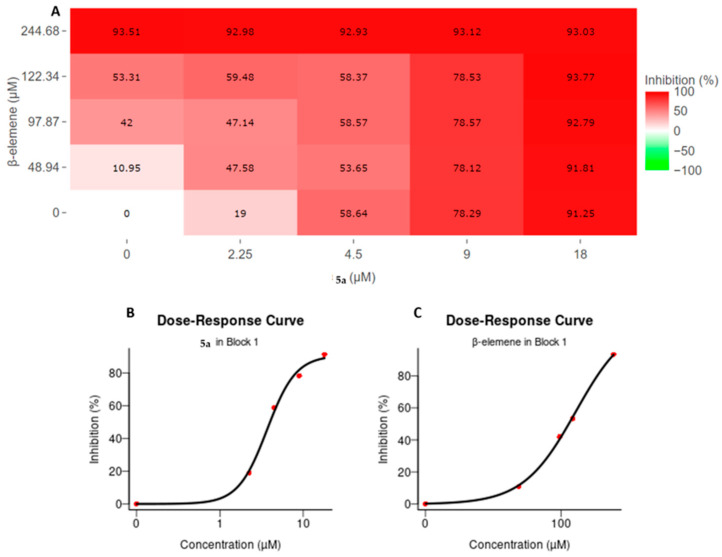
Cellular inhibition profile of β-elemene and compound **5a** in the context of 4 × 4 matrix dose treatment. (**A**) Inhibitory activities of compounds on cell viability by themselves and in combination. MV4-11 cells were incubated with combinations of compounds for 72 h and viability measurements were performed. Viability percentages compared to control cells were calculated and analyzed in the SynergyFinder tool. Increasing red color represents increasing inhibition value; (**B**,**C**) represent the dose–response curve for compound **5a** and β-elemene, respectively. The corresponding percent inhibition of each dose is shown with red dots; (**B**) Compound **5a**; (**C**) β-elemene.

**Figure 2:**
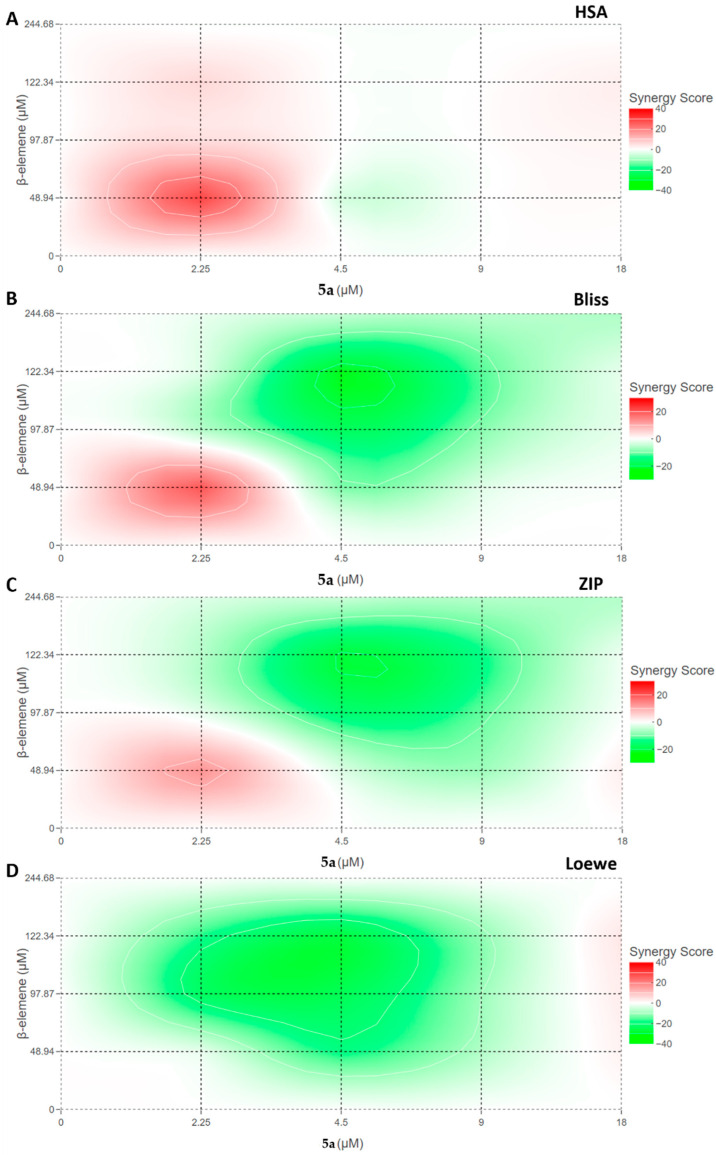
Synergism analysis of β-elemene and compound **5a** with different interaction models. The inhibition values obtained after combinations of four different doses of both compounds were analyzed in the Synergyfinder tool. Red areas represent increased synergy of combinations. The color barometer rates the variation between −40 and 40. (**A**) HSA (**B**) Bliss (**C**) ZIP (**D**) Loewe.

A 4 × 4 dose matrix analysis was used to assess the cellular inhibitory efficacy of β-elemene and compound **5a** in combinational treatments. In our previous studies, it was determined that the IC_50_ value of β-elemene on MV4-11 cells was 25 µg/mL and compound **5a** was 4.5 µM. Based on this, the dose matrix analysis was performed by combining the IC_50_, upper, and lower doses of the compounds. The doses were 10, 20, 25, and 50 µg/mL for β-elemene and 2.25, 4.5, 9, and 18 µM for compound **5a**. Cells were treated with these doses and combinations for 72 hours. At the end of 72 h, cellular viability analyses were performed with the WST-1 test, and absorbance values were calculated relative to untreated control cells. The obtained viability values were analyzed with the SynergyFinder tool. β-elemene doses were entered into the software as a µM equivalent of µg/mL to perform analysis in the SynergyFinder tool. Figure 1A shows the percentages of cellular inhibition of the respective doses and their combinations after compound treatments. In addition, dose response curves for both compounds are shown in Figure 1B for compound **5a** and in Figure 2C for β-elemene respectively. As expected, treatments with 50 µg/mL β-elemene and 18 µM compound **5a**, which were 100% inhibitory, resulted in over 90% cell death in all combinational manners. The 9 µM compound **5a** treatment showed a 78% inhibition efficiency in itself and other combinations. The 25 µg/mL β-elemene treatment, which is the IC_50_ dose for β-elemene, while its efficacy was 53.31%, showed inhibition in the range of 55–60% in combinations of 2.25 and 4.5 µM compound **5a**. The situation was similar for the IC_50_ dosing of compound **5a**. While it showed a 58.64% inhibition on its own, inhibition was around 58% in combinational treatments. No potentiating effect was observed in the combination of the IC_50_ values of the two compounds. The 2.25 µM compound **5a** treatment showed a 19% inhibition by itself, while combinational doses with β-elemene showed increased efficacy. This increase was found to be 47% and above. Additionally, inhibition was 10.95% with the β-elemene 10 µg/mL dose, while with the 2.25 µM compound **5a**, this value increased by 36.63% to reach 47.58%. In summary, all these results profiled the combinatorial cellular inhibition phenotype of β-elemene and compound **5a**.

### 2.2. Synergism Analysis of β-Elemene and Compound **5a**

Using the potency of these two compounds, synergy analyses were performed to determine which combination would be used to modulate potential signaling pathways in MV4-11 cells. Many theories have been put forward from the past to the present in the applications carried out with the use of different compounds for certain targets. Although it is desired to determine a conclusion such as antagonism, non-interaction, and synergism for the interaction of drugs, this issue is still not fully settled. As Tang et al. asserted, the analysis of each combination should be evaluated against its pharmacology [28]. Therefore, in our study, we performed synergy analyses for β-elemene and compound **5a** by using models of four different hypotheses with the Synergyfinder tool. Thus, we aimed to determine both the interaction mechanism of these two drugs and the optimal dose combination for further studies. Figure 2 shows the synergism profiles between β-elemene and compound **5a** according to four different synergy models: HSA (highest single agent), Bliss, ZIP (zero interaction potency), and Loewe. According to these models, the interaction between two drugs is evaluated mathematically as follows. It is classes as antagonism if the synergy score is below −10, additive if it is between −10 and 10, and synergism if it is higher than 10. In this study, the mean synergy scores of the models were calculated as 2.35 for HSA, −5.22 for Bliss, −5.31 for ZIP, and −8.31 for Loewe, respectively. Considering the obtained scores, we can say that there is no antagonism or synergy between β-elemene and compound **5a**. Although the scores show that the relationship is additive, a closer look at the diagrams of the models reveals increased efficacy and synergy scores in combinations of 2.25 µM of compound **5a** with different doses of β-elemene, especially in the HSA model. Our previous research has aimed to examine the detailed mechanistic properties of the two compounds in MV4-11 cells. After our studies centered on a 25-µg/mL β-elemene dose and a 9-µM compound **5a** dose, we aimed to make further analyses in this study by combining 25 µg/mL of β-elemene with 2.25 µM of compound **5a**. We thought that these two combinations would contribute to the modulation of potential signaling pathways, with a cellular inhibition efficiency of 59.48%. 

### 2.3. Cell Morphology Analysis

**Figure 3:**
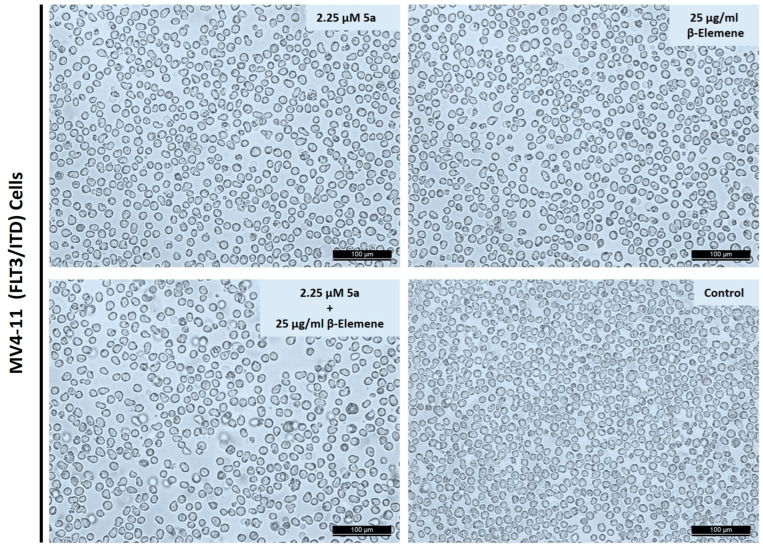
Cell morphology observations after compounds or combination treatments. Cells were incubated compound **5a** or β-elemene or in combination for 72 h. Randomly selected areas were photographed under an inverted microscope with 100× magnification. Scale bar represents 100 µm.

Cell morphology analyses were performed to observe the phenotypic effects of selected doses on MV4-11 cells. Cells were treated 24 h after seeding with 2 µM of compound **5a** or 25 µg/mL of β-elemene, or a combination of these two doses. An amount of 0.1% DMSO was used as a control. Cells were photographed after 72 h of treatments. As seen in Figure 3, no toxic death was observed in the cells, as expected in single compound applications. The presence of more selective and programmed inhibition was confirmed by intact membrane structures, preservation of cell integrity, and the near absence of dead cell debris. A similar morphology was obtained at the combination dose. Thus, both single doses and combinational applications showed that selective inhibition continued after 72 h of application in cells.

### 2.4. Multiple Protein Profiling to Analyze Alterations of Cell Death Targets 

**Figure 4:**
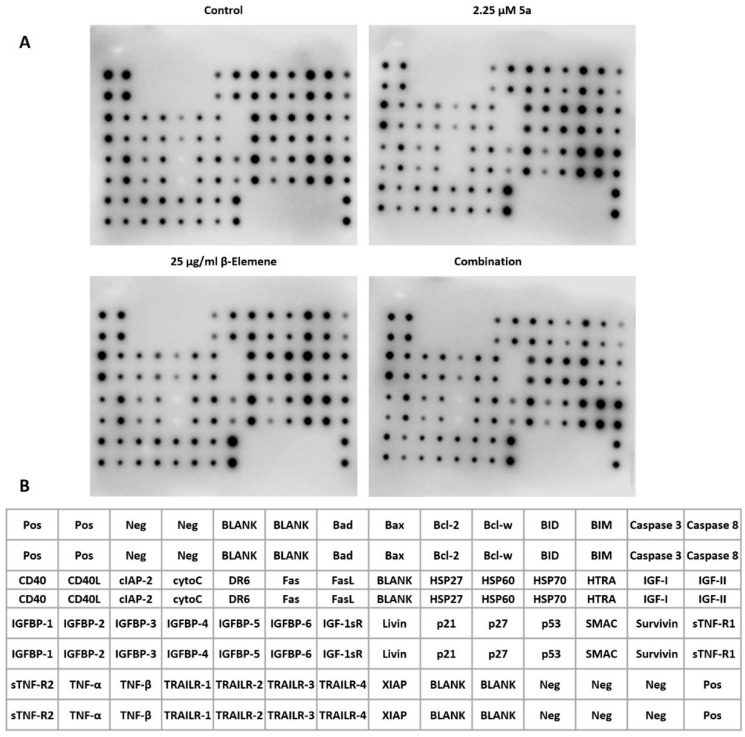
Multiple protein profiling with human apoptosis antibody array. (**A**) Chemiluminescence images obtained after 72 h of treatment of MV4-11 cells (**B**) Map of proteins on the array. Pos stands for positive control and is used for normalization. Neg means negative control and contains antibody dilution buffer. Blank are empty spots. Other spots represent the relevant protein.

**Figure 5:**
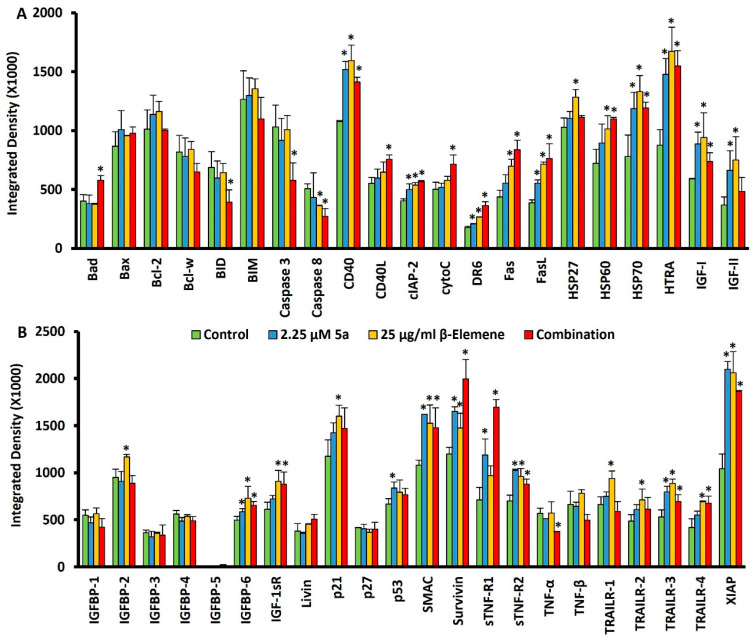
Quantitative analysis of multiple protein profiling. (**A**) Quantification of proteins between Bad and IGF-II from the array map (**B**) Quantification of proteins between IGFBP-1 and XIAP from the array map * *p* < 0.05.

After the analyses we performed with the combinatorial approach, a protein-level-based array analysis was performed to investigate how the selected combination affected the cell death pathways. As a result of treatment of the cells with oxindole compound at a dose of 9 µM for 48 h in our previous research; upregulated proteins were identified as Bad, HSP27, HSP60, HSP70, HTRA, Livin, p27, and TNFβ, and downregulated proteins as Caspase8, IGFBP-2, IGFBP-4, and Survivin [14]. On the other hand, in another study focusing on β-elemene, MV4-11 cells were treated with 25 µg/mL β-elemene for 48 h and protein measurements were performed. In β-elemene-mediated protein changes; upregulated targets were identified as Bcl-2, Bcl-w, BID, CD40, FasL, HSP27, HSP60, HSP70, HTRA, IGF-I, IGFBP-2, Livin, p21, p27, p53, SMAC, and Survivin. There was no protein that was significantly downregulated [25]. These targets, modulated by these two compounds, formed the basis of this study. We sought an answer to the question of whether we could increase the advantages or reduce the disadvantages with a rational combination of these two compounds. For this purpose, we wanted to supplement 25 µg/mL β-elemene with 2.25 µM compound 5a. We considered the main axis compound as β-elemene and aimed to fortify it with an oxindole derivative. MV4-11 cells were treated with a combination of 25 µg/mL β-Elemene and 2.25 µM compound **5a** for 72 h. Then, proteins from four individual groups (untreated control, β-elemene treated, **5a**-treated, and combination-treated) were isolated, and simultaneous immunoblotting analysis of 43 individual cell-death-associated proteins was performed using a Human Apoptosis Antibody Array kit (Abcam, Cambridge, United Kingdom). Figure 4 represents the chemiluminescence images (Figure 4A) and array map (Figure 4B). Figure 5A,B show the values obtained as a result of the quantification of proteins. When we looked at the proteins in the Bad-BIM range (Bad, Bax, Bcl-2, Bcl-w, BID, BIM) we observed that BAD expression increased, and BID expression decreased significantly with the combination. Notable in this range was the stabilization of the anti-apoptotic Bcl-2 and Bcl-w, which tend to increase with individual compounds. When we consider our previous report and this study’s data together, we can say that the resistance side brought by β-elemene in this axis is broken by compound **5a.** On the other hand, interestingly, Casp3 and Casp8 expression decreased significantly with the combination. This indicates that the combination altered equilibria in the apoptotic machinery compared to the singular treatment. One of the striking modulations occurred in CD40 and CD40L. Only the combination dose significantly increased CD40L, while CD40 was elevated in all three treatments. We can say that the combination contributes to the ligand activity, and accordingly, CD40 activity increases with the induction of β-elemene. Cyto-C was among the targets that increased only with the combination treatment. Although this increase does not seem to contribute to caspase cascade activation, it comes to mind that it may be increased as a result of the suppression of Bcl-2 family members by combination. DR6 increased after 72 h of treatment with both compounds, accompanied by a higher level of combination treatment. While Fas and its ligand (FasL) did not change in our previous studies, in this study, FasL increased with all three treatments, the highest in the combination. Fas was also similarly increased for β-elemene and combination but not **5a**. All three of the heat shock proteins had increased for the two compounds in our previous studies. In this study, it was seen that lower dose **5a** did not cause an increase in HSP27 and HSP60. It was also observed that the β-elemene-mediated increase in HSP27 decreased with the combination. Increases in HSP70 were noticeable in all three treatments. It was noted that 2 µM **5a** tried to deal with these stress proteins. HTRA levels were characterized by a tremendous increase for all three treatments. Even though the levels of IGF-I and IGF-II, which have the ability to inhibit apoptosis, increased with the treatments, the combination attempted to bring them down. While no change was observed in 1, 3, and 4 of the IGFBP1-6 series, an increase was observed in 2.25 **5a** with β-elemene, as expected, and consistent with our previous study. But it was remarkable that this was brought down with the combination. Compound **5a** underwent this reduction significantly at the 9 µM dose, where 2.25 µM supplementation reduced the induction of β-elemene. Although the story behind this is complex, we think it may be related to the reduction in IGF-II. No significant, combination-related changes were observed in Livin, p21, p27, and p53. While SMAC contributed positively to the apoptosis machinery with an increase in all three treatment types, Survivin also increased and negatively influenced it. The substantial rise observed in the combined treatment for Survivin demonstrated that the two drugs exhibited this target-specific synergy. Soluble tumor necrosis factor receptors 1 (sTNF-R1) and 2 (sTNF-R2) were significantly increased with combination treatment, especially for sTNF-R1. On the other hand, while there was no change in TNF-β, there was a decrease in TNF-α only in the combination. All the TRAIL death receptor series (TRAILR 1–4) showed significant increases with 72 h of 25 µg/mL β-elemene treatment. The fact that it increased both apoptosis mediators, TRAILR1 and TRAILR2, and decoy receptors, TRAILR3 and TRAILR4, is somewhat difficult to explain. Finally, XIAP protein increased significantly with all three treatments and showed an inhibitory effect on the apoptosis process. Considering all these massive change dynamics, 25 µg/mL β-elemene combined with 2.25 µM **5a** caused regulations in various cell death targets. Table 1 summarizes selected proteins that were significantly changed by this combination.

## 3. Discussion

Our earlier studies have shown that β-elemene and **5a** targeted FLT3-ITD in AML cells and demonstrated strong inhibition of proliferation [14,25]. Therefore, we hypothesized that combining β-elemene and **5a** could provide a strong synergistic effect against FLT3-ITD-mutated AML cells. FLT3 mutations comprise the current therapy under the banner of drug resistance. Blocking FLT3-mutated oncoproteins will disrupt growth factor-mediated signaling cascades. The pharmacological synergism of anticancer drugs has been utilized in clinical settings and has produced promising results [29]. The previous reports demonstrated that combination therapy against AML could provide strong synergistic inhibition of proliferation [30]. The pharmacological synergism enhances the therapeutic effect, decreases drug resistance and tumor growth, and inhibits the metastatic potential of monotherapy. Using the combination to enhance the therapeutic effect was a main objective of the investigation. Different combinations of drugs targeting AML have demonstrated different synergistic effects in earlier studies [30,31]. To investigate the synergistic effects of compounds, **5a** and β-elemene, we used models based on many hypotheses with the synergy finder tool [28,32,33]. Our dose matrix results showed that the IC_50_ of β-elemene and compound **5a** was not changed by the combination. These observations suggested that the combination of compound **5a** and β-elemene demonstrated no potentiating cellular inhibition. We found that the effects of β elemene and **5a** were additive without any sign of antagonism (Figure 2). Using a combination of drugs against AML has demonstrated promising results in earlier studies [34]. As the mutations in the FLT3 in AML pose significant problems with the current drug therapies, it is crucial to investigate alternative means of therapy that can target the mutated FLT3 oncoproteins.

We also performed morphological experiments to confirm that targeting mutated FLT3 expressing cells. We observed that the cell membrane was intact post-treatment, meaning these drugs modulated the apoptotic pathways. These findings indicate that drugs act in apoptotic ways. The earlier results also reported that β-elemene promotes apoptosis in various cell lines [35,36,37]. To investigate the molecular induction and alteration of various signaling proteins that are involved in cell death and cancer cell proliferation by these drugs, we performed a protein-level array. We observed that the apoptotic protein BAD expression was upregulated significantly, meaning that the exposure combination of β-elemene and compound **5a** activates the apoptotic pathways. BAD, an antagonist of BCL-2, promotes apoptosis and decreases cell proliferation [38]. Although we also observed that the expression of BID, caspase 3, and caspase 8 was downregulated, the reason may be the alteration of apoptotic machinery and decreased drug resistance in the FLT3 mutated AML cells [39]. Various reports reveal that significant upregulation of various pro-apoptotic proteins leads to drug resistance [40]. CD40 and CD40L play major roles in cancer and cell death pathways, so it was important to investigate their expression status [41]. In earlier studies, it has been reported that CD40L enhances apoptosis in drug-resistant cancer [42]. Furthermore, we found that the expression status of CD40L was not significantly changed in our previous results when used as monotherapy; however, the expression of CD40L was significantly high in the combination, but no change was found when exposed individually. These results suggest that a combination of alteration of the CD40L expression status and disruption of the signaling pathways might be responsible for the apoptosis effect. The change in the expression balance between CD40 and CD40L may be another reason for promoting programmed cell death. It was deciphered earlier that ligation of CD40 leads to cancer cell death. The increased expression of CD40L means that the combination of β-elemene and compound **5a** increases the ligation of CD40, causing the AML cells to die [43]. MAPKs are a group of kinases that regulate various physiological processes, such as cellular dynamics [44]. Reports have claimed that blocking CD40 has disrupted the MAPK and PI3K pathways involved in cell survival [43]. From earlier investigations, β-elemene has also shown anticancer effects by blocking MAPK-PI3K-mTOR pathways [45]. These findings reveal the anticancer effect of β-elemene by blocking cancer cell survival pathways. The FILT3 mutations increase the drug resistance to PI3K inhibitors, so blocking FLT3 could overcome the resistance and show promising anticancer responses [46]. Cytochrome c is a marker of apoptosis; we observed a significant increase in its expression in combination treatment, although we could not observe any increase in individual treatments [47]. The upregulation of cytochrome-c, the prominent cell death marker, is a promising outcome from the combination treatment. The death receptor 6 (DR6) was highly upregulated, supporting the evidence that the combination treatment promotes cell death. It is clear from the earlier results that DR6 is actively involved in the apoptosis of cells [48]. Several drugs demonstrate anticancer activity by upregulating DR6 [49]. FasL, the main apoptosis marker, was significantly upregulated on combination treatment. It has been demonstrated earlier that various chemotherapeutic drugs promote the expression of FasL [49]. These results suggest that the combination treatment alters key protein targets that directly or indirectly promote AML death via apoptosis.

Coming to other proteins indirectly involved in regulating apoptosis, we found that HSP27, IGFBP-2, Livin, p21, p53, and IGF-II were not changed on combination treatment. However, sTNF-R1 was significantly upregulated on combination treatment. The upregulation of TNF by the combination treatment indicates the activation of the TNF superclass of the death receptor family [50]. In earlier studies, TNF, FasL, and other related TNFs have been extensively studied for their role in apoptosis [50]. Upregulating the expression of sTNF-R1 and FasL signifies that they promote the combination treatment block of FLT3-mediated oncogenic signaling, ultimately leading to upregulation key apoptotic signaling cascades. HTRA, a potential target in cancer treatment because of its role in the programmed cell death pathways, was highly upregulated in individual and combination treatments [51]. The increased levels of HTRA also signify an increased response to chemotherapy, which is a promising sign against FLT3 drug-resistant AML cancers [51]. The release of SMAC into the cytoplasm, a mitochondrial protein signifying its role in apoptosis, was also found to be significantly upregulated in individual and combination treatments, which supports the notion that there is key involvement of the mitochondrial protein in addition to cytochrome-c [52]. Survivin, considered a molecular biomarker in AML, was significantly upregulated, signifying that some protein crosstalk might have influenced its expression levels [53]. TRIAL death receptors, which play a significant role in apoptotic pathways and get upregulated on drug treatment or during the process of apoptosis, were found to be highly upregulated [54]. It has been reported that TRIAL receptors are highly sensitive to synthetic drugs and natural products [54]. 

The FLT3-ITD mutations in the AML make the cells resistant to anticancer drugs because multiple pathways are involved in cancer cell survival, like the STAT5-MAPK-AKT axis, which plays an essential role in crosstalk with multiple cell survival proteins. The activation of these pathways leads to cancer cell proliferation and anti-apoptotic protein upregulation. It took 20 years for researchers to understand the FLT3 mutations. Only three FLT3-based, clinically approved drugs are currently being used in AML subjects. Still, the mechanisms of FLT3 resistance are not clear because of the complexity of the signaling pathways that are being activated [55]. The main limitation of our findings is that the experiments were conducted in vitro only, and these results need to be reproduced in vivo models. Most importantly, we could not perform studies in knock-out and knock-in model cell lines to determine the exact mechanisms responsible for the anticancer effect. Therefore, it is important to investigate the mechanistic insights to better design our therapeutics for managing AML. Furthermore, future studies should be focused on the effectiveness, potency, and applications in relapse situations that could be performed in vivo and in clinical studies. However, more findings are required to understand the phenomenon of increased expression despite the increased expression of key apoptotic markers. From these observations, there are valid findings that support the notion that combination of β-elemene and compound **5a** significantly enhanced their therapeutic potential by increasing the activation of various key targets involved in AML cell death.

## 4. Materials and Methods

### 4.1. Compounds

**Figure 6:**
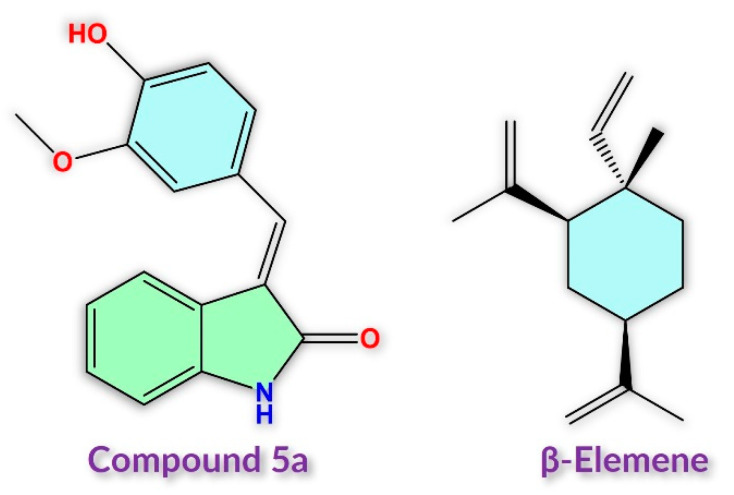
Chemical structures of compound **5a** and β-elemene used in the study.

The compound **5a** (3-(4-Hydroxy-3-methoxybenzylidene)indolin-2-One) (InChI=1S/C16H13NO3/c1-20-15-9-10(6-7-14(15)18)8-12-11-4-2-3-5-13(11)17-16(12)19/h2-9,18H,1H3,(H,17,19)) was synthesized as we have previously described [14]. β-elemene (≥98.0% purity) ((1S,2S,4R)-1-Ethenyl-1-methyl-2,4-bis(1-methyl ethenyl)cyclohexane, (1S,2S,4R)-(−)-2,4-Diisopropenyl-1-methyl-1-vinyl cyclohexane) (InChI = 1S/C15H24/c1-7-15(6)9-8-13(11(2)3)10-14(15)12(4)5/h7,13-14H,1-2,4,8-10H2,3,5-6H3/t13-,14+,15-/m1/s1) was purchased from Sigma (Cat no: 63965, St. Louis, MO, USA), with a molecular formula of C_15_H_24_ and molecular weight of 204.35. Alternatively, stock solutions, compound **5a** and β-Elemene (Figure 6), were dissolved in dimethyl sulfoxide (DMSO) (Sigma, St. Louis, MO, USA) at final concentrations of 20 mM and 25 mg/mL, respectively.

### 4.2. Cells and Culture Conditions

The FLT3-expressing and ITD-mutant acute myeloid leukemia cell line MV4-11 (Cat. no. CRL-9591) was purchased from the American Type Culture Collection (ATCC, Maryland, MI, USA). MV4-11 cells were cultured in Iscove’s Modified Dulbecco’s Medium (Cat. no. 01-058-1A, Biological Industries, Haemek, Israel) supplemented with 10% heat-inactivated fetal bovine serum (Biowest, Nuaillé, France), 100 U/mL penicillin, and 100 µg/mL streptomycin (Gibco, Waltham, MA, USA) and 2.5 µg/mL plasmocin (Invivogen, Toulouse, France). Cells were routinely cultured in cell culture flasks at 37 °C in a humidified standard incubator with 5% CO_2_ and subcultured when they reached 70–80% confluence. Before starting the main experiments, cells were counted with a hemocytometer, and viability of cellular populations was determined using the trypan blue dye (Gibco, Waltham, MA, USA) exclusion method [56].

### 4.3. Drug Combination Assay

A drug combination assay was performed to evaluate the synergistic potential of β-elemene and compound **5a** combinations on MV4-11 cells. Cells were treated with different doses of β-elemene and compound **5a** with a 4 × 4 dose matrix above and below their IC_50s_ which were selected in accordance with our previous studies [14,25]. First, MV4-11 cells were seeded in transparent 96-well plates with 1.0 × 10^4^ cells/well and cultured for 24 h. Doses were created with serial dilution from the main stocks of β-elemene or compound **5a**. For matrix combinations, β-elemene was studied at 50 µg/mL, 25 µg/mL, 20 µg/mL, 10 µg/mL and, compound **5a** was studied at 18 µM, 9 µM, 4.5 µM and 2.25 µM. 24 h after seeding, compound sets were applied to the cells triplicate, and incubated for 72 h at 37 °C with 5% CO_2_. At the end of the incubation period, WST-1 reagent (TaKaRa Bio., Shiga, Japan) was added to each well, and plates were incubated for 3 h under the conditions mentioned above. Then, the absorbance of each well was measured with a microplate reader (Infinite^®^ 200 PRO) (Tecan Life Sciences, Männedorf, Switzerland) at a wavelength of 450 nm and 600 nm (as the reference). After that, the recorded absorbance values were used to calculate the percentages of cell viability and inhibition. The data were analyzed with the SynergyFinder tool [32], and synergy scores were generated using reference synergy models [33,57].

### 4.4. Observation of Cell Morphology

Analysis of cellular morphology after treatments was performed as previously described [58]. In brief, MV4-11 cells were seeded in 6-well culture plates with a cell density of 5.0 × 10^5^ cells/well. After 24 h of incubation, cells were treated with β-elemene (25 µg/mL), compound **5a** (2.25 µM), and a combination dose of 25 µg/mL of β-elemene and 2.25 µM of compound **5a**. As a negative control, a medium containing 0.1% DMSO was used. Cells were incubated with compounds for 72 hours at 37 °C and 5% CO_2_. Subsequently, cells were photographed for morphological change assessment under an inverted microscope, a Leica DM IL LED with a DFC-290 camera (Leica, Wetzlar, Germany) [59].

### 4.5. Human Apoptosis Antibody Array

A Human Apoptosis Antibody Array kit (Abcam, Cambridge, United Kingdom) was used to detect related genes in the human-apoptosis-signaling pathway, as we have previously described [14,25]. MV4-11 cells were seeded at a density of 5 × 10^5^ cells/well in a 6-well plate for simultaneous detection of 43 human apoptotic marker concentrations in cell lysates. At the end of 24 h of incubation, cells were exposed to β-elemene (25 µg/mL), compound **5a** (2.25 µM), and a combination of compound **5a** (2.25 µM) and β-elemene (20 µg/mL) for 72 h. As a negative control, 0.1% DMSO-containing growth medium was used. At the end of treatment, cell pellets were lysed in 400 µL 1× lysis buffer and incubated at 4 °C for 30 min with gentle shaking. The lysates were then spun down for 10 min at 4 °C at 14,000 rpm and supernatant was collected. A Pierce BCA Protein Assay Kit (Thermo Fisher Scientific, Waltham, MA, USA) was used to quantitatively measure protein concentrations [60]. Antibody array membranes were blocked for 30 min at room temperature with 1× blocking buffer. Membranes were incubated overnight at 4 °C with cell lysates, which is diluted with 1.2 mL of 1× blocking Buffer. Then, the membranes were thoroughly washed with wash buffer I and wash buffer II, and then 1× biotin-conjugated anti-cytokines were added and incubated at 4 °C overnight. After several extensive washing steps using buffers described above, the membranes were incubated with 1× HRP-Streptavidin for 2 h at room temperature. Finally, membranes were washed again, and to enhance visualization, detection buffers C and D were used. The images were captured using a chemiluminescence Odyssey^®^ XF Imaging System (LI-COR Biosciences, Lincoln, NE, USA). The intensity score of each array spot was measured with the ImageJ software program (National Institutes of Health, Bethesda, MD, USA), and the following formula was used to normalize the expression of individual proteins in each membrane: X(Ny) = X(y) ∗ P1/P(y), P1 = mean signal density of positive control spots on the reference Array, P(y) = mean signal density of positive control spots on Array “y”, X(y) = mean signal density for spot “X” on array for sample “y”, X(Ny) = normalized signal intensity for spot “X” on Array “y”.

### 4.6. Statistical Analysis

The statistical significance between experimental groups were calculated using Student’s *t*-test. *p*-values below 0.05 were considered statistically significant.

## 5. Conclusions

Current therapy against AML faces many challenges, like drug resistance, because of the FLT3 tyrosine kinase receptor mutations. Identifying new molecules targeting the mutated FLT3 AML cancers is crucial based our previous studies on β-elemene and compound **5a**. We demonstrated that both drugs showed strong inhibition in the proliferation of FLT3-mutated AML cells. The current study demonstrated that the combination of β-elemene and compound **5a** induces pharmacological inhibition of FLT3-mutated signalling and inhibits AML cell proliferation without antagonizing each other. Several important proteins were modulated via combination treatment, signifying the importance of using a combination as an approach against aggressive AML cancers. We propose that these results be validated in in vivo models so that this combination can be used with AML patients in the future.

## Figures and Tables

**Table 1:** Selected targets and directions that positively contribute to programmed cell death after 72 h treatments to MV4-11 cells.

Target Name	2.25 µM Compound 5a	25 µg/mL β-Elemene	Combination
Bad	NC	NC	↑
CD40	↑	↑	↑
CD40 ligand	NC	NC	↑
CytoC	NC	NC	↑
DR6	↑	↑	↑
Fas	NC	↑	↑
Fas ligand	↑	↑	↑
HSP27	NC	↑	NC
IGFBP-2	NC	↑	NC
IGF-II	↑	↑	NC
sTNF-R1	↑	NC	↑

NC: Not changed ↑: Upregulated.

## Data Availability

Available with the corresponding author on request.
